# The effect of different methods of intravenous injection on glass particle contamination from ampules

**DOI:** 10.1186/s40064-015-1632-0

**Published:** 2016-01-06

**Authors:** Ga Eul Joo, Kyeong-Yae Sohng, Michael Yong Park

**Affiliations:** College of Nursing, The Catholic University of Korea, 222 Banpo-daero, Seocho-gu, Seoul, 06591 Republic of Korea; Department of Radiology, Seoul St. Mary’s Hospital, The Catholic University of Korea, Seoul, Republic of Korea

## Abstract

There have been many studies on glass particle contamination from glass ampules during the injection of glass ampules, but only the contamination from direct IV bolus injection has been measured. This research aimed to study the difference in glass particle contamination from ampules with different intravenous administration methods commonly used in clinical practice. Four methods were studied: IV bolus injection directly after immediate aspiration, IV bolus injection directly after 2 min’ delayed aspiration, IV bolus injection directly after aspiration with a filter needle, and side shooting to an infusion set with an in-line filter. 45 ampules per method for a total of 180 ampules were used. The number and length of glass particles were measured using a slide scanner. Aspiration was performed without specifically using a slow aspiration method. The longest glass particle was observed in the immediate aspiration group. The side shooting group showed the lowest maximum number of glass particles per ampule. The side shooting group also showed the smallest number of glass particles, but it was statistically insignificant. Using a filter needle syringe and 2 min’ delayed aspiration, which are frequently recommended to minimize contamination, may not be as effective as commonly believed, unless combined with a slow and low pressure aspiration method. Using a side shooting to an infusion set with an in-line filter may minimize glass particle contamination from ampules even without a slow and low pressure aspiration method, but more evidence from a larger study is needed.

## Background

Medication is an important and basic duty for health care professionals. It is the responsibility of a nurse to administrate drugs safely and accurately to the patient. Many drugs are manufactured as a glass ampule due to its sterility and portability, and often premeasured as a single dose. However, glass particles may be introduced during the manufacturing, opening, and injection of glass ampules (Caudron et al. 2011). These particles can cause various harmful side effects when they circulate in the body. Injected glass particles can travel through the blood vessels to arrive at various organs, and cause inflammatory responses. They are known to cause blockages, embolism, tissue necrosis, and sepsis (Brewer and Dunning 1947; Shaw and Lyall 1985; Carbone-Traber and Shanks 1986; Preston and Hegadoren 2004). In spite of these risks, glass ampules are commonly used for injections.

There are several strategies to decrease glass particle contamination when using glass ampules. Filter needle syringes have been introduced into practice and are recommended for special patient care, such as neonatal patient care to maximize patient safety (Heiss-Harris and Verklan 2005). The use of prefilled syringe drugs may also be a useful method to prevent glass particle contamination in peripheral intravenous admixtures (Yorioka et al. 2006). It has been recommended that wiping glass ampules with alcohol before opening should be a routine part of neuraxial anaesthesia to reduce the contamination of glass ampules (Hemingway et al. 2007). Glass or inorganic particulate contaminants may be reduced by opening glass ampules using a vacuum machine, especially small-sized particles which may not be removed with membrane filters (Lee et al. 2011). The American Society of Health-system Pharmacists (ASHP) guidelines recommend that 5 µm filter straws or filter needles be used when aspirating the contents of ampules to minimize particulate contamination (ASHP guidelines on compounding sterile preparations 2014). The policies regarding the use of filter needles vary between institutions and countries, and filter needles are often not used because they are expensive and cumbersome. There have been many prior studies of glass particle contamination during the opening of glass ampules. The main focus of these studies was the side effect of glass particles in the body or the degree of glass particle contamination. The degree of glass ampule particle contamination has been studied based on the ampule size and the opening method of glass ampules, the size of injection needles, and the use of filters (Caudron et al. 2011; Shaw and Lyall 1985; Carbone-Traber and Shanks 1986; Preston and Hegadoren 2004; Heiss-Harris and Verklan 2005; Turco and Davis 1972; Purdie and Punchihewa 1982; Pinnock 1984).

Prior studies used a microscope to measure glass particles in a slide or a paper sampled from a syringe aspirated from opened glass ampules. These studies estimated glass particles introduced into the body when a single dose of a glass ampule is injected directly. However, there are other commonly used methods to inject drugs into the vein, such as direct IV bolus injection, side shooting into the Y-shape injection port of the infusion set with in-line filter, and infusion after mixing the drug with other IV solutions, as well as using an intermittent infusion device such as injection adapter caps. For inpatients, side shooting using a Y-shape infusion set or infusion after mixing the drug with other IV solution is more frequently used than direct IV bolus injection. Y-shape infusion sets for side shooting commonly contain a 15 µm filter at the end of the set, but filters of various sizes are used in different hospitals and departments. Some counties recommend aspirating drugs 1–2 min after opening a glass ampule to settle down glass particles [The Korea Food and Drug Administration (KFDA) 2010].

However, there have been few studies to verify the effectiveness and feasibility of these methods in clinical practice. This study compares the difference in glass particle contamination from ampules with different intravenous administration methods used in clinical practice.

## Methods

### Research design

The study was a descriptive observational study which collected, measured, and analyzed the degree of glass particle contamination from glass ampules with different intravenous injection methods. Four injection methods were used in this study: IV bolus injection directly after immediate aspiration, IV bolus injection directly after 2 min’ delayed aspiration, IV bolus injection directly after aspiration with a filter needle syringe, and side shooting to an infusion set with an in-line filter.

### Sample

A total of 45 ampules per injection method resulting in a total of 180 ampules were opened and analyzed for this study.

45 ampules per injection method were chosen by the distribution analysis of medium effect size of 0.25, level of significance of 0.05, and statistical power of 0.80 with the G* Power 3.0 program. Glass ampules of 2 mL ascorbic acid were used from a single manufacturer.

### Materials used

1. Syringe
  Unfiltered and filtered 3 ml syringes with 23G needles were used. The filter pore size of the filtered needles was 5 μm.
2. Infusion set
  A general Y-shaped infusion set with a 15 μm pore in-line filter attached at the end of the set was used. The ampule contents were injected through the proximal Y-shaped connection (Fig. 1).

**Fig. 1:**
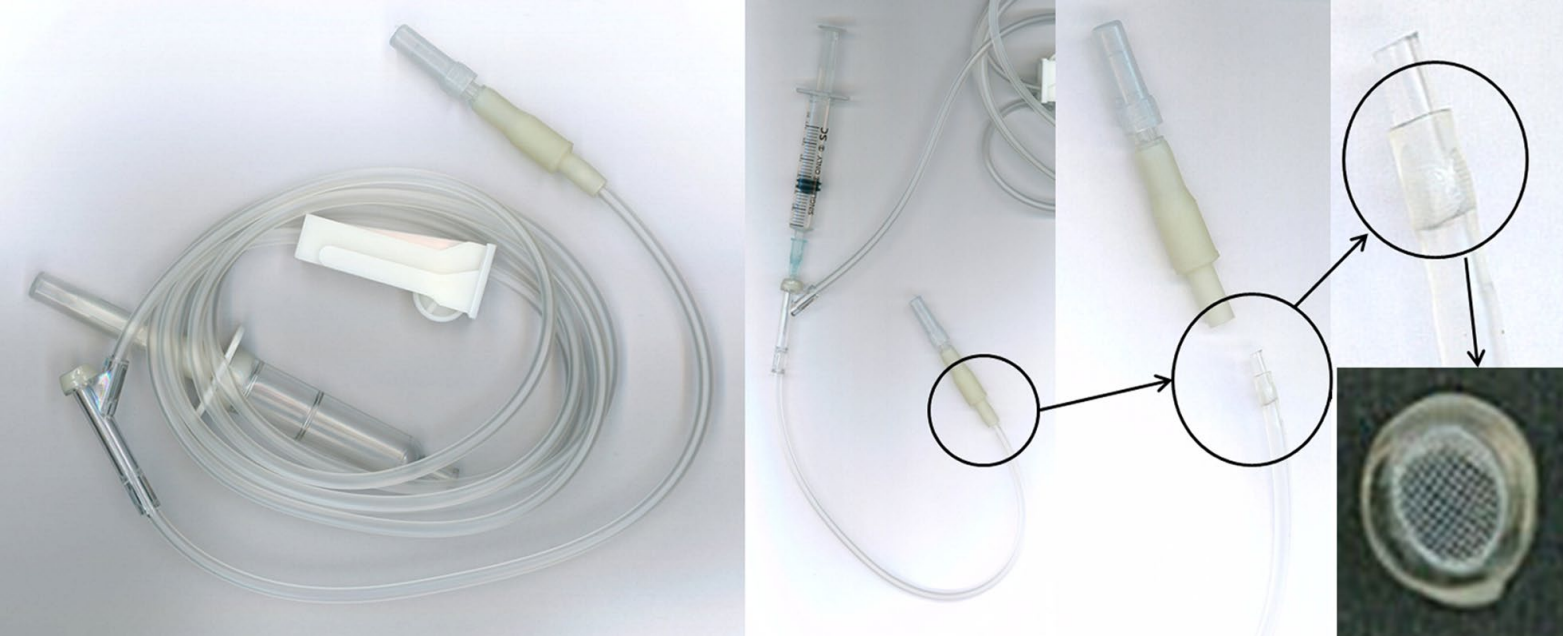
Infusion set with an in-line filter. Figure shows a general Y shaped infusion set. After injection into the Y shaped connection, the injection fluid flows through a 15 μm pore in-line filter at the distal end (*magnified circle* in figure)
3. Micro centrifuge
  A high speed micro centrifuge (Micro 12, Hanil Science Industrial, Incheon, Korea) was used for the research.
4. Slide Scanner
  A slide scanner (Pannoramic MIDI, 3DHISTECH Ltd., Budapest, Hungry) with an attached camera with manually adjustable magnifying power and auto focus capabilities (Allied Vision Technologies, Stadtroda, Germany) was used.
5. Others
  Other materials used for the research were disposable alcohol swabs, microscope slides, microscope cover glasses, wipers, micropipette, sterilized pipette tips, and sterilized microtubes.

### Procedure

**Fig. 2:**
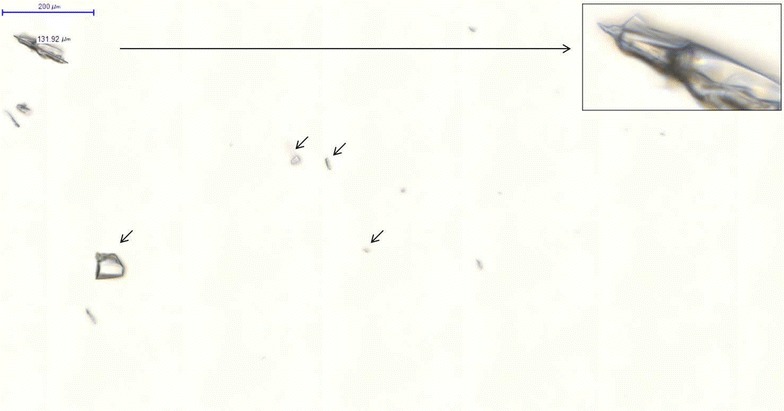
Image from the slide scanner of aspirated fluid from group 1 (immediate aspiration), showing a large glass particle magnified (*long arrow*) with several smaller glass particles (*short arrows*)

1. Opening of the ampule
  After the neck of the ampule was cleaned with disposable alcohol swabs twice, the ampule was opened by breaking the neck along the scored line with an outward snapping motion.
2. Aspiration of the ampule
  The ampule was aspirated by inserting a needle into the ampule slowly and carefully to avoid touching the edge of the ampule opening. The ampule was completely aspirated (2 mL) by tilting the ampule gradually to accumulate the drug at the lower portion of the ampule. Aspiration was performed without specifically using a slow aspiration method by a nurse with over 10 years of clinical experience.
3. Group assignment
  Ampules were assigned to one of the following four groups randomly by the random number generation function of Microsoft Excel:
  IV bolus injection directly after immediate aspiration group (group 1):
    Aspirated with an unfiltered syringe immediately after opening an ampule.
  IV bolus injection directly after 2 min’ delayed aspiration group (group 2):
    Aspirated with an unfiltered syringe 2 min after opening a glass ampule to allow glass particles to settle onto the bottom of an ampule.
  IV bolus injection directly after aspiration with a filter needle syringe group (group 3):
    Aspirated with a filter needle syringe immediately after opening an ampule.
  Side shooting to an infusion set with an in-line filter group (group 4):
    Injected at the Y-shape injection port of an infusion sets (side shooting) following aspiration immediately after opening an ampule.
4. Slide preparation
  Aspirated drug from each ampule was injected into a sterilized microtube, and high speed centrifugation was performed to precipitate glass particles to the bottom of the microtube. They were each sampled by 20 µL, using a micropipette and disposal pipette tips. The 20 µL sample which contained glass particles was infused onto a slide glass and covered by a cover glass.
5. Scanning of slides
  One slide per ampule was made resulting in a total of 180 slides and the slides were scanned by a slide scanner to obtain magnified images. Suitable range of magnifying power was obtained by using the manually-adjustable magnifying power capability of the camera, and then the slides were scanned by using the auto-focus capability of the camera.
6. Measuring of glass particles
  Images obtained by the scanner were analyzed by a specialized slide viewing software (Pannoramic Viewer, version 1.15). The length of glass particles in a randomly selected area (6000 µm × 6000 µm) of each slide was measured by electronic calipers and the number of glass particles of each length range was tabulated (Fig. 2). They were grouped into nine groups according to the length of glass particle (≤5, 5–10, 10–15, 15–50, 50–100, 100–150, 150–200, 200–300, and >300 µm). This was because the pore diameter in a filter needle syringe was 5 µm, the diameter of a pulmonary capillary was 10 µm, and the pore diameter of an in-line filter at the end of an infusion set was 15 µm.

### Data analysis

The collected data was analyzed by using the statistical program SPSS version 19.0. The differences of the four intravenous injection methods were measured by Kruskal–Wallis test and χ^2^-test after log transformation of the number and the length of glass particles.

### Ethical considerations

The research was reviewed and authorized by the Institutional Review Board (IRB No: MC12QISI0177). Before collecting and analyzing the materials, the researcher was trained at the Integrative Research Support Center for the operation of micro centrifuge, slide scanner and slide viewing software. During the training, the researcher conducted more than 10 pilot tests including slide preparation, scanning the slide and measuring of glass particles.

## Results

### Number of glass particles with different intravenous injection methods

**Fig. 3:**
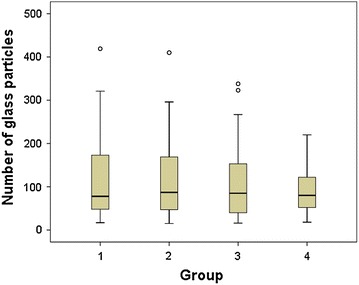
Box plot of number of glass particles per ampule by group. *Group 1* Bolus IV after immediate aspiration. *Group 2* Bolus IV after 2 min’ delayed aspiration. *Group 3* Bolus IV after aspiration with a filter needle syringe. *Group 4* Side shooting to an infusion set with an in-line filter

The total number of glass particles in the 180 glass ampules tested was 19,473. The number of glass particles in each glass ampule was between 15 and 419 particles and the average number of glass particles in each glass ampule was 108.18 ± 79.45. Among 19,473 glass particles in 180 glass ampules tested, there were 5169 glass particles in group 1 (immediate aspiration), 5311 glass particles in group 2 (2 min delay), 4971 glass particles in group 3 (filtered needle), and 4022 glass particles in group 4 (side shooting). The number of glass particles was the smallest in group 4 (side shooting), but the differences were not statistically significant. There were glass ampules containing more than 400 glass particles in group 1 (immediate aspiration) and group 2 (2 min delay), while all ampules in group 4 (side shooting) contained less than or equal to 220 glass particles (Table 1, Fig. 3).

**Table 1:** Number of glass particles per ampule (*n* = 180)

Group	Number			*χ* ^*2*^	*p*
	Mean ± SD	Median	Range		
1 (n = 45)	114.87 ± 89.26	78.00	17–419	0.647	0.886
2 (n = 45)	118.02 ± 90.60	87.00	15–410		
3 (n = 45)	110.47 ± 81.58	85.00	16–338		
4 (n = 45)	89.38 ± 48.35	80.00	18–220		
Total	108.18 ± 79.45	83.50	15–419		

*Group 1* IV bolus injection directly after immediate aspiration, *Group 2* IV bolus injection directly after 2 min’ delayed aspiration, *Group 3* IV bolus injection directly after aspiration with a filter needle syringe and *Group 4* Side shooting to an infusion set with an in-line filter

Ampules were grouped by the number of glass particles contaminated; less than 50, between 51 and 100, between 101 and 150, between 151 and 200, between 201 and 300, and more than 301 glass particles. The number of ampules by the number of glass particles between groups did not show a statistically significant difference (Table 2). The number of ampules with more than 100 glass particles was the smallest in group 4 (side shooting).

**Table 2:** Number of ampules by number of glass particles (*n* = 180)

Group	Number of glass particles						*χ* ^*2*^	*p*
	≤50	51–100	101–150	151–200	201–300	>300		
1	13 (28.9)	13 (28.9)	6 (13.3)	5 (11.1)	6 (13.3)	2 (4.4)	11.14	0.743
2	12 (26.7)	13 (28.9)	6 (13.3)	7 (15.6)	6 (13.3)	1 (2.2)		
3	14 (31.1)	13 (28.9)	4 (8.9)	7 (15.6)	5 (11.1)	2 (4.4)		
4	10 (22.2)	19 (42.2)	10 (22.2)	4 (8.9)	2 (4.4)	0 (0.0)		
Total	49 (27.2)	58 (32.2)	26 (14.4)	23 (12.8)	19 (10.6)	5 (2.8)		

*Group 1* IV bolus injection directly after immediate aspiration, *Group 2* IV bolus injection directly after 2 min’ delayed aspiration, *Group 3* IV bolus injection directly after aspiration with a filter needle syringe and *Group 4* Side shooting to an infusion set with an in-line filter

### Length of glass particles with different intravenous injection methods

**Fig. 4:**
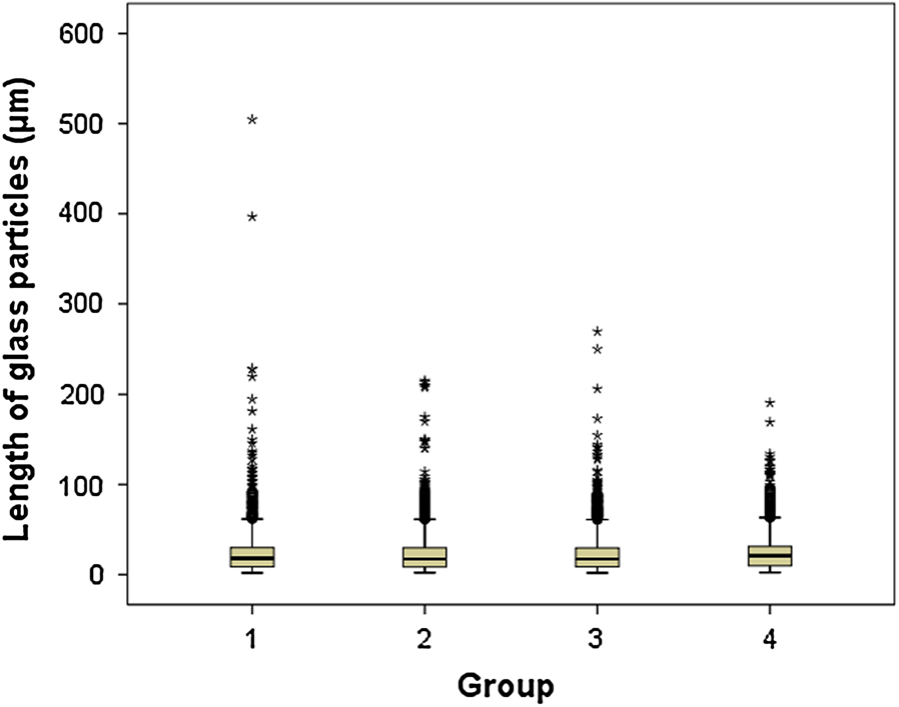
Boxplot of length of glass particles by group. *Group 1* Bolus IV after immediate aspiration. *Group 2* Bolus IV after 2 min’ delayed aspiration. *Group 3* Bolus IV after aspiration with a filter needle syringe. *Group 4* Side shooting to an infusion set with an in-line filter

The average length of glass particles was 21.38 ± 17.06 µm and the range of glass particle length was between 1.92 and 504.67 µm. The longest glass particle was observed in group 1 (immediate aspiration). Glass particles longer than 300 µm were observed in group 1 (immediate aspiration), but all glass particles in group 4 (side shooting) were shorter than 191 µm (Table 3; Fig. 4).

**Table 3:** Length of glass particles (*n* = 19,473)

Group	Length (µm)			*χ* ^*2*^	*p*
	Mean ± SD	Median	range		
1 (n = 5169)	21.27 ± 18.55	18.06	1.97–504.67	0.826	0.843
2 (n = 5311)	20.87 ± 16.60	17.36	2.11–215.25		
3 (n = 4971)	20.97 ± 16.68	17.36	1.92–269.29		
4 (n = 4022)	22.70 ± 16.04	20.83	2.39–190.67		
Total	21.38 ± 17.06	18.34	1.92–504.67		

*Group 1* IV bolus injection directly after immediate aspiration, *Group 2* IV bolus injection directly after 2 min’ delayed aspiration, *Group 3* IV bolus injection directly after aspiration with a filter needle syringe and *Group 4* Side shooting to an infusion set with an in-line filter

The number of glass particles longer than 10 µm, longer than 50 µm, and longer than 100 µm were 70.4, 3.8 and 0.4 %, respectively. There was a significant difference in the number of particles when grouping by the length of glass particles between the different intravenous injection methods (Table 4). Glass particles longer than 150 µm were observed less in group 3 (filtered needle) and group 4 (side shooting).

**Table 4:** Number of glass particles by range of their length (*n* = 19,473)

Group	Length of glass particles (μm)									χ^2^	*p*
	≤5	5–10	10–15	15–50	50–100	100–150	150–200	200–300	>300		
1 (n = 5169)	390 (7.5)	1167 (22.6)	702 (13.6)	2732 (52.9)	153 (3.0)	17 (0.3)	3 (0.1)	3 (0.1)	2 (0.0)	91.93	0.000
2 (n = 5311)	465 (8.8)	1191 (22.4)	741 (14.0)	2727 (51.3)	166 (3.1)	13 (0.2)	3 (0.1)	5 (0.1)	0 (0.0)		
3 (n = 4971)	404 (8.1)	1109 (22.3)	724 (14.6)	2548 (51.3)	164 (3.3)	17 (0.3)	2 (0.0)	3 (0.1)	0 (0.0)		
4 (n = 4022)	200 (5.0)	830 (20.6)	533 (13.3)	2278 (56.6)	164 (4.1)	15 (0.4)	2 (0.0)	0 (0.0)	0 (0.0)		
Total	1459 (7.5)	4297 (22.1)	2700 (13.9)	10,285 (52.8)	647 (3.3)	62 (0.3)	10 (0.1)	11 (0.1)	2 (0.0)		

*Group 1* IV bolus injection directly after immediate aspiration, *Group 2* IV bolus injection directly after 2 min’ delayed aspiration, *Group 3* IV bolus injection directly after aspiration with a filter needle syringe and *Group 4* Side shooting to an infusion set with an in-line filter

## Discussion

This study analyzed the contamination of glass particles with different intravenous injection methods commonly used in clinical practice. For inpatients, an intravenous injection using a side shooting to an infusion set is more frequently used than a direct IV bolus injection, but prior studies analyzed the contamination of glass particles using only the direct IV bolus injection method. The filter needle syringe method is recommended for use in intensive care units and patients that are severely ill, such as infants, cancer patients, and major surgical patients. This study also included the 2 min’ delayed aspiration method since the guideline of some countries recommended the 2 min’ delayed aspiration method [The Korea Food and Drug Administration (KFDA) 2010].

The number of glass particles was the smallest in group 4 (side shooting), but it was not statistically significant. This is different from the results of prior studies which suggested that the number of glass particles was reduced significantly with an in-line filter (Shaw and Lyall 1985; Preston and Hegadoren 2004; Heiss-Harris and Verklan 2005; Pinnock 1984; Sabon et al. 1989; Oie and Kamiya 2005). This difference may be explained by the result of Carbone-Traber and Shanks study (Carbone-Traber and Shanks 1986), which found that it is important to aspirate fluid into a syringe slowly and by a low pressure injection method when using not only a filter needle syringe but also a side shooting to an infusion set with an in-line filter. Our study did not use any specific method to slowly aspirate ampules, and may have used a faster speed of aspiration resulting in increased aspiration pressure compared with prior studies. This is an important point, as many nurses will be aspirating at a rapid speed in a busy clinical environment. Further research for the optimal speed, amount, and pressure of aspiration to minimize particle contamination from glass ampules may be needed.

There were ampules with more than 400 glass particles in group 1 (immediate aspiration) and group 2 (2 min delay), but less than 220 glass particles in all ampules in group 4 (side shooting). This suggests that the contamination of an unusually large number of glass particles may be prevented by using an infusion set with an in-line filter.

The number of glass particles in group 2 (2 min delay) was similar to that of group 1 (immediate aspiration) in our research. This may be due to the glass particles settled in the bottom of the ampule being aspirated into the syringe because the ampule was inclined during aspiration. An ampule contains sufficient drug fluid, so it is sometimes recommended not to incline an ampule when aspirating the fluid [The Korea Food and Drug Administration (KFDA) 2010]. But in clinical practice, the ampule is often inverted or tilted sideways with all the drug fluid in the ampule being aspirated into the syringe to minimize the loss of drug fluid (Craven and Hirnle 2009). Therefore, further research to find out how much fluid should be aspirated to prevent aspirating glass particles settled down on the bottom of the ampule is needed. This suggests that further education of nurses may be needed to avoid inclining ampules when aspirating drug fluids.

In this study, the length of the shortest glass particle and the longest glass particle was 1.92 and 504.67 µm, respectively. The longest glass particle was observed in group 1 (immediate aspiration). This can be explained by long glass particles in group 2 (2 min delay) settling down to the bottom of ampules due to their weight, and filters in group 3 (filtered needle) and group 4 (side shooting) filtering out long glass particles. Since the diameter of pulmonary vasculature is less than 10 μm, they may cause harmful side effects to patients in the medium to long term.

The average length of glass particles was the shortest in group 2 (2 min delay), but it was statistically insignificant. This may be because the long glass particles had time to settle down onto the bottom of an ampule due to their weight, as stated before. Therefore, to prevent the injection of abnormally long glass particles into the body, it may be useful to utilize natural precipitation by waiting a certain period of time before aspirating drug fluid from an ampule after opening the ampule. However, further research is required to determine whether it is safe to wait after opening an ampule in terms of contamination control, whether it is possible at busy clinical practice situations, and the effectiveness in terms of the number of glass particles.

The absolute number of glass particles by their length was the smallest in group 4 (side shooting), but the average length of glass particles was the shortest in group 2 (2 min delay) among the four groups. No glass particles longer than 200 μm were found only in group 4 (side shooting). This suggests that the in-line filter effectively filtered the injection of very long glass particles.

**Fig. 5:**
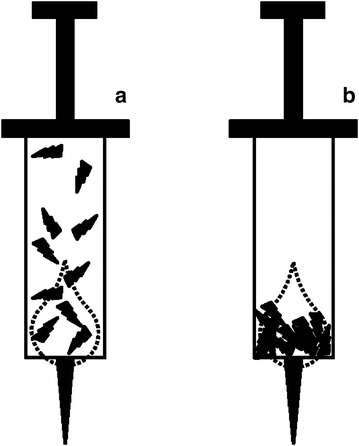
Sampling method. **a** Prior studies sampled a portion of an ampule with irregularly distributed glass particles. **b** This study used the sediment of precipitated particles from a centrifuged ampule resulting in sampling of the glass particles of whole ampule

The significance of this study is that the number and the length of glass particles were measured and analyzed in an injection administration environment similar to that of actual clinical practice. In most prior studies, glass particles in one or two drops of potentially irregularly distributed fluid of an ampule were measured (Preston and Hegadoren 2004; Hemingway et al. 2007). In this study, the entire drug fluid of an ampule contaminated by glass particles during opening was centrifuged to precipitate and a slide was created with sediment (Fig. 5), resulting in more glass particles than in prior studies where only a small portion of the drug fluid was used.

Although most glass particles were easily observed due to their double refraction, small glass particles around 5 µm in lengths were too small to be analyzed in some slides. This may be due to the potential air gaps between a slide glass and a cover glass which reduced the clarity of the microscope. These slides were re-measured with an increased magnification power by using the manually-adjustable magnifying power capability of the camera. Accordingly, ambiguous glass particles in the image of initial scanning were reconfirmed by rescanning them with a different magnification power. There were a total of 10 slides which were re-measured.

Aspiration at a faster rate than prior studies (Carbone-Traber and Shanks 1986) may have been another factor that contributed to the increase in the number of glass particles compared to prior studies. In a busy clinical practice, aspiration is probably often performed faster than what is recommended.

In prior studies, the syringe used was irrigated to include the glass particles possibly attached to the syringe walls (Carbone-Traber and Shanks 1986; Lee et al. 2011). However, in our study, they were excluded since the glass particles attached inside of a syringe in clinical practice would prevent them from being injected into the patient. It is possible that some glass particles may have attached and remained in the micropipette tips and microtubes, suggesting potential underestimation of glass particles in the slides, which suggests the possibility of an even greater risk of glass particles in real clinical practice.

## Conclusion

Glass particle contamination of an ampule occurred with all intravenous injection methods and there are risks of harmful side effects to patients due to this contamination. Although 95 % of glass particles less than 20 μm disappeared within 1 year by becoming dissolved in animal clinical studies, it is possible for a large amount of small glass particles to accumulate and cause adverse reactions (Brewer and Dunning 1947).

This study showed that the 2 min’ delayed aspiration method, which is recommended in the guidelines of some countries [The Korea Food and Drug Administration (KFDA) 2010], was not effective in reducing the injection of the total number of glass particles in clinical practice despite preventing the injection of exceptionally large glass particles. This study suggests that IV bolus injection directly after 2 min’ delayed aspiration and filter needle syringes, which are frequently recommended to minimize contamination, may not be as effective as commonly believed, unless they are combined with a slow and low pressure aspiration method. A side shooting to an infusion set with an in-line filter may prevent injection of an irregularly large number of particles and filter out exceptionally long glass particles from ampules even with a faster aspiration speed, but more evidence from a larger study is needed.
